# The Possible Role of IVIG in the Treatment of Atrial Fibrillation Accompanied by Fulminant Myocarditis in a 12-Year-Old Pediatric Patient

**DOI:** 10.1155/2021/6689865

**Published:** 2021-05-20

**Authors:** Can Yilmaz Yozgat, Osman Yesilbas, Selcuk Uzuner, Nigar Bayramova, Gokce Ergun, Lala Nurmammadova, Eser Tekin, Hafize Otcu Temur, Yilmaz Yozgat

**Affiliations:** ^1^Faculty of Medicine, Bezmialem Vakif University, Istanbul, Turkey; ^2^Department of Pediatric Critical Care Medicine, Karadeniz Technical University, Trabzon, Turkey; ^3^Department of Pediatrics, Bezmialem Vakif University, Istanbul, Turkey; ^4^Department of Radiology, Bezmialem Vakif University, Istanbul, Turkey; ^5^Department of Pediatric Cardiology, Istanbul Medipol University, Istanbul, Turkey

## Abstract

**Background:**

Fulminant myocarditis (FM) is a potentially lethal condition in children due to rapid progressive hemodynamic instability and cardiogenic shock. Patients with FM might show different clinical manifestations on emergency department admission.

**Case:**

Herein, we describe the case of a 12-year-old girl who was admitted to our institution's emergency department due to complaints of abdominal pain and incessant vomiting. However, we detected an early onset of atrial fibrillation (AF) accompanied by FM. The patient's condition of AF and severe hemodynamic disorder was successfully treated in our institution's pediatric intensive care unit.

**Conclusion:**

To the best of our knowledge, this is the first report of the co-occurrence of FM and AF successfully treated in childhood. This case report will serve as a guide for the treatment of cases with FM accompanied by AF.

## 1. Introduction

Myocarditis is an inflammatory disease that manifests in the myocardium of the heart [1]. The condition is generally caused by prevalent viral infections, which may lead to myocardial injury or virally mediated immune responses [2]. Myocarditis can be categorized into two groups: nonfulminant or fulminant. The categorization is performed in terms of clinical, echocardiographic, histological, and hemodynamic findings [3]. Fulminant myocarditis (FM) with acute onset can cause fetal ventricular arrhythmia and severe hemodynamic instability emerging with cardiogenic shock with multiple-organ dysfunction and sudden death. All of these conditions require immediate hemodynamic support [4]. In our search of the literature on this subject, we did not identify any report about the development of early atrial fibrillation (AF) presenting with fulminant myocarditis (FM). In this case, the condition in a pediatric patient was further complicated by cardiogenic shock.

## 2. Case Report

**Figure 1:**
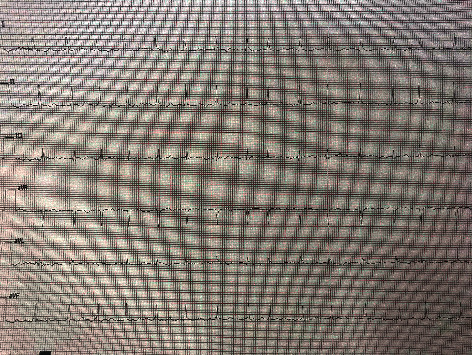
ECG showing atrial fibrillation.

A 12-year-old female was admitted to our hospital emergency department (ED) with abdominal pain and incessant vomiting. Her physical examination and blood results were normal. Transabdominal ultrasound results were normal as well, and the ED team decided to discharge the patient from the ED with a prediagnosis of acute gastroenteritis and referred her to the pediatrics department for further management. However, a day later, the patient was again admitted to the ED with more complaints of abdominal pain, headache, and chest pain. All of the patient's complaints mentioned above had also progressed in severity. The second physical examination in the ED revealed low blood pressure, and ECG results showed signs of arrhythmia. The patient consulted the Department of Pediatric Cardiology to determine the main reason for the arrhythmia findings. The patient's general condition was lethargic, and her blood pressure was 80/37 mmHg. Her ECG findings were indicative of atrial fibrillation (Figure 1).

**Figure 2:**
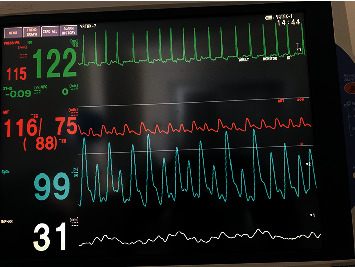
The bedside monitoring image showing AF-induced polymorphic artery trace, and the alterations in pulse oximeter were in accordance with polymorphic artery trace.

A decision was made by our multidisciplinary team, including critical care, cardiology, and emergency medicine, to transfer the patient to the pediatric intensive care unit (PICU) for a higher level of care. Initial blood results at PICU admission revealed hemoglobin (HGB) of 10 g/dL, white blood cells (WBCs) of 11 × 10^3^/*μ*mm^3^, thrombocytopenia (139 × 10^3^/*μ*mm^3^), high C-reactive protein (CRP) of 45 mg/L and a high erythrocyte sedimentation rate (ESR) of 35 mm/hr. The first analyses of cardiac enzymes revealed troponin-I 1100 IU/mL (normal range 0–40), creatine kinase 450 IU/mL (normal range 22- 198), creatine kinase (CK-MB) isoenzyme 150 IU/L (normal range 5-10), amylase 185 gr/dL (normal range 30-110), and albumin 2.8 gr/dL (normal range 3.4–5.4). Serum electrolytes were unremarkable. The patient was put under strict monitoring. Bedside imaging showed AF-induced polymorphic artery tracing. The alterations in pulse oximetry were in accordance with the polymorphic artery trace (Figure 2). An echocardiographic inspection revealed severe dilation of the left side of the heart, and severe mitral insufficiency was detected, with substantial systolic dysfunction (ejection fraction (EF) 40% and shortening fraction (SF) 20%). Additionally, the patient was prescribed adrenaline, milrinone, furosemide, and carnitine for her severe heart failure. Although the patient's left atrium was enlarged, thrombosis inside the left atrium was not detected. At this point, it had been more than 72 hours since the patient's first ED admission with her initial complaints of abdominal pain and incessant vomiting. Thus, cardioversion was not performed due to the probability of microthrombus formation. Warfarin treatment was promptly initiated. The thyroid hormone profile was unremarkable. Cardiac magnetic resonance imaging (MRI) was performed to explain the etiology, which resembled acute myocarditis due to the existence of T2-weighted edema images. The patient was prescribed an IVIG infusion of 1 gr/kg IV for two days in a row.

**Figure 3:**
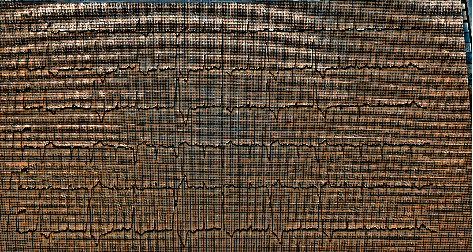
ECG disclosing AF and multifocal ventricular ectopic beats due to the aberrant conduction of atrial fibrillation.

**Figure 4:**
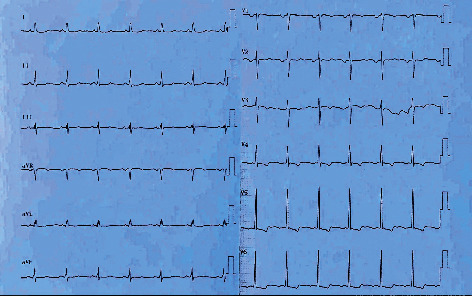
ECG strip showing after cardioversion. The patient's rhymes were turned into sinus.

**Figure 5:**
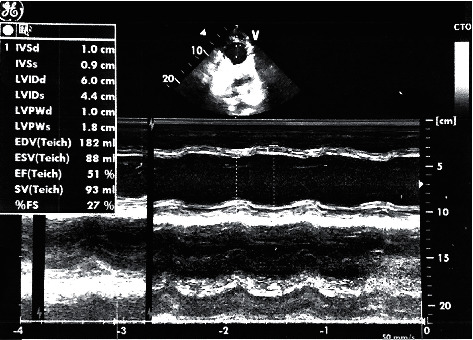
M-mode LV function before the patient was discharged from the PICU.

The patient's condition was discussed in a council composed of two pediatric critical care specialists and two pediatric cardiologists. The council decided that the extracorporeal membrane oxygenation unit (ECMO) should be prepared if the event that the patient developed sudden cardiac arrest or her blood pressure suddenly dropped. After the administration of the second dose of IVIG in the PICU, the patient's blood pressure was mildly elevated (90/60/70 mmHg), and a small amelioration of ejection fraction 44% and a shortening fraction of 22% were noted on echocardiography. On the 3^rd^ day in the PICU, there were no signs of atrial fibrillation improvement; however, multifocal ventricular ectopic beats were detected due to the aberrant cardiac conduction of atrial fibrillation (Figure 3). There was no significant improvement or any notable development in the clinical situation of the patient throughout her stay between the 3^rd^ day and 15^th^ day in the PICU. On the 15^th^ day in the PICU, a transesophageal echocardiography examination was performed, and no signs of thrombosis in the left atrium were observed. The patient was started on amiodarone of 200 mg/day. On the 3^rd^ day of amiodarone treatment, cardioversion was performed at a dose of 0.5 Joules/kg in a total of 30 Joules. The rhythms became a sinus rhythm (Figure 4). The multidisciplinary team continued both treatments of amiodarone and warfarin for two weeks, and the patient's general condition improved significantly during that time. On the 30^th^ day of PICU admission, the patient's blood pressure was within normal range, and an echocardiographic inspection revealed significant improvement in an EF of 50% and a SF of 26% compared with her first day of PICU admission (Figure 5). The patient was discharged from the hospital on the 35^th^ day after her first admission to the ED. The patient continues to be attended at our pediatric cardiology clinic monthly and is still under treatment with amiodarone.

## 3. Discussion

Fulminant myocarditis has a rapidly progressive clinical manifestation: severe hemodynamic instability, cardiogenic shock with multiple organ dysfunction syndromes, and sudden death [4]. Wang et al. reported a meta-analysis of myocarditis in seven studies (158 FM patients and 388 NFM patients) [5]. According to their analysis, the FM group was detected to have much lower systolic blood pressure (SBP), higher creatine kinase (CK) levels, more extensive QRS duration, lower left ventricular ejection fraction (LVEF), thicker left ventricular posterior wall diameter (LVPWd), higher incidence of ST depression, ventricular tachycardia/ventricular fibrillation (VT/VF), and syncope. Our case was considered to be FM because of the significantly lower SBP, higher creatine kinase (CK) level, lower LVEF, and AF. The patient had an initial complaint of abdominal pain and chest pain. The main reason for her first complaint of abdominal pain might have been mesenteric ischemia, and the patient might also have developed chest pain due to impaired coronary perfusion. Our diagnosis of acute myocarditis was based on the clinical characteristics and findings of noninvasive tests. The results were achieved by echocardiography, ECG, and MRI. Cardiac troponin, CKMB, and CRP levels are all indicators of a possible myocardial injury. Currently, MRI is considered the gold standard due to its noninvasive utility in diagnosing and evaluating myocarditis in children [6]. The Lake Louise criteria were formulated in 2009. The requirements describe the findings on contrast-enhanced MRI, increasing the sensitivity and specificity for the diagnosis of myocarditis. The results can indicate the possibility of focal or global myocardial edema, hyperemia and capillary leakage, and fibrosis or necrosis seen as late gadolinium enhancement [7]. T2-weighted edema imaging has become a reliable way to detect acute myocardial inflammation, and T2-weighted edema imaging was carried out for our case.

Electrocardiographic changes are common clinical manifestations of viral myocarditis. The changes include sinus bradycardia, ventricular tachycardia/ventricular fibrillation, ST-T changes, AV blocks, ventricular premature beats, and sinus node dysfunction. Atrial fibrillation rarely manifests in children with acute myocarditis [8]. There are no reported cases of FM or coexistence with AF in children. The main reason for the lack of reports about AF in the literature might be that it is incredibly arduous to diagnose AF, especially in children. In the present case, the patient's admission to the ED occurred due to abdominal pain and the detection of AF. During her stay in the PICU, the AF resolved after a series of cardioversions were performed. The patient's general condition became stable as well. Since 1990, two significant pediatric hospitals in Boston and Los Angeles in the U.S. have added the treatment of a high dose of IVIG for suspected cases of acute myocarditis to their routine protocols [9]. Dilawar et al. performed a study on how to achieve an adequate response to acute viral myocarditis with the treatment of high-dose IVIG and steroids in childhood [10].

Acute myocarditis can be harmful to the myocardium due to the mediation of predominant immunological mechanisms rather than by the effects of viral infection and replication. A few studies have shown that IVIG treatment can be effective in the amelioration of LVEF. Fulminant myocarditis is defined by its deleterious effects such as acute hemodynamic collapse, fatal arrhythmias, or low cardiac output [11]. IVIG has multifactorial effects. IVIG has proven to have not only antiviral effects, though also anti-inflammatory effects by suppressing inflammatory cytokines and neutralizing pathogens [12, 13]. During IVIG therapy, the plasma levels of IL-10, IL-1 receptor antagonist, and soluble tumor necrosis factor receptors (sTNF-Rs) were elevated crucially [13, 14]. Moreover, the reduction of oxidative stress by IVIG therapy was also observed [13]. Therefore, IVIG was supposed to relieve the negative inotropic effects by inhibiting the proinflammatory cytokines [14].

To our knowledge, this is the first case reported in the literature of the early onset of AF presenting with FM complicated by cardiogenic shock in a pediatric patient.

## 4. Conclusion

Pediatric patients who have FM should be investigated for the possibility of AF based on ECG, and cardioversion should be performed at the most suitable time. Patients with fulminant myocarditis (FM) can be admitted to the ED for different complaints due to the development of severe hemodynamic instability. In particular, pediatric patients might show deterioration in their general condition. Early atrial fibrillation and abdominal pain may be the first clinical features of FM.

## Data Availability

All data used to support the findings of this study are available upon request to the corresponding author.
